# Age-Related Decline of Sensorimotor Integration Influences Resting-State Functional Brain Connectivity

**DOI:** 10.3390/brainsci10120966

**Published:** 2020-12-10

**Authors:** Natsue Yoshimura, Hayato Tsuda, Domenico Aquino, Atsushi Takagi, Yousuke Ogata, Yasuharu Koike, Ludovico Minati

**Affiliations:** 1 Institute of Innovative Research, Tokyo Institute of Technology, 4259 Nagatsuta, Midori-ku, Yokohama 226-8503, Japan; tsuda@cns.pi.titech.ac.jp (H.T.); atsushi.takagi.yx@hco.ntt.co.jp (A.T.); y.ogata1110@gmail.com (Y.O.); koike@pi.titech.ac.jp (Y.K.); minati.l.aa@m.titech.ac.jp (L.M.); 2 Neural Information Analysis Laboratories, ATR, Kyoto 619-0288, Japan; 3 Integrative Brain Imaging Center, National Center of Neurology and Psychiatry, Kodaira, Tokyo 187-8551, Japan; 4 PRESTO, JST, Kawaguchi, Saitama 332-0012, Japan; 5 Department of Neuroradiology, Fondazione IRCCS Instituto Neurologico Carlo Besta, 20133 Milano, Italy; Domenico.Aquino@istituto-besta.it; 6 NTT Communication Science Laboratories, Atsugi, Kanagawa 243-0198, Japan; 7 Center for Mind/Brain Science (CIMeC), University of Trento, 38123 Mattarello, TN, Italy

**Keywords:** arm-reaching task, functional connectivity, kinesthesia, motor coordination, normal aging, proprioception, resting-state fMRI, sensorimotor integration

## Abstract

Age-related decline in sensorimotor integration involves both peripheral and central components related to proprioception and kinesthesia. To explore the role of cortical motor networks, we investigated the association between resting-state functional connectivity and a gap-detection angle measured during an arm-reaching task. Four region pairs, namely the left primary sensory area with the left primary motor area (S1left–M1left), the left supplementary motor area with M1left (SMAleft–M1left), the left pre-supplementary motor area with SMAleft (preSMAleft–SMAleft), and the right pre-supplementary motor area with the right premotor area (preSMAright–PMdright), showed significant age-by-gap detection ability interactions in connectivity in the form of opposite-sign correlations with gap detection ability between younger and older participants. Morphometry and tractography analyses did not reveal corresponding structural effects. These results suggest that the impact of aging on sensorimotor integration at the cortical level may be tracked by resting-state brain activity and is primarily functional, rather than structural. From the observation of opposite-sign correlations, we hypothesize that in aging, a “low-level” motor system may hyper-engage unsuccessfully, its dysfunction possibly being compensated by a “high-level” motor system, wherein stronger connectivity predicts higher gap-detection performance. This hypothesis should be tested in future neuroimaging and clinical studies.

## 1. Introduction

It is well-established that sensorimotor performance gradually declines with normal ageing, which eventually leads to a pressing clinical problem due to the resulting reduced mobility together with increased incidence of falls and other injuries [1,2,3,4]. Such a reduction reflects a multitude of complex processes, involving both anatomical and physiological changes at the peripheral level, that is, musculoskeletal, perfusional and neuromuscular degradation, together with incipient dysfunction within the central nervous system itself, namely altered feedback and regulation between the motor regions, as well as cognitive factors [5,6,7,8,9,10,11,12,13,14]. With this landscape of changes, the gradual hinderance of proprioception, namely the perception of the body or limbs’ position and forces in space, and kinesthesia, the corresponding notion for dynamics, has also been demonstrated to significantly contribute to falls and other accidents [15]. Multiple brain regions are related to proprioception and kinesthesia, including cortical motor areas such as the supplementary motor area (SMA) and the premotor cortex (PM) [16]. In particular, postural sway and balance control in older adults have been related to central proprioceptive processing via parietal, frontal, and insular cortical areas as well as via the basal ganglia [17,18,19,20]. A comprehensive review of this field can be found in [21].

As in other areas of neuroimaging, a question remains as to whether the identified associations may depend on experimental design [22]. This limitation could be alleviated through recourse to resting-state functional magnetic resonance imaging (rsfMRI), which probes intrinsic connectivity without active engagement in a task. Using this method, not only can the neural representations of cognitive and motor functions, including their changes during healthy aging, be addressed [23], but also individual behavioral performance of working memory, memory capacity, perceptual performance, or learning ability can be predicted [24,25,26,27]. In clinical scenarios such as preoperative mapping, rsfMRI has recently gained increased recognition due to the fact that it is convenient to deploy, not requiring any additional patient compliance beyond that necessary for conventional structural imaging, and it does not depend on task design, active engagement and performance level, more directly indexing intrinsic aspects of brain function and physiology than task-based assessment [28,29].

Based on a working hypothesis that the decline in sensorimotor integration with aging, with a particular emphasis on proprioception and kinesthesia, involves the sensorimotor brain networks; in this exploratory study, we apply an arm-reaching task to identify some resting-state functional connectivity (rsFC) elements delineating an interaction between age and gap detection ability during the task.

## 2. Materials and Methods

### 2.1. Participants

Twenty right-handed healthy participants, 10 younger (age 24 ± 3 years, all male) and 10 older (62 ± 3 years, 3 male) participated in this experiment. The study protocol was approved by the ethics committee, “Human Subjects Research Ethics Review Committee” of the Tokyo Institute of Technology (approval no. 2,017,142 of Mar. 30, 2018, P.I. N.Y) and carried out in accordance with the Declaration of Helsinki.

### 2.2. Arm-Reaching Task

**Figure 1:**
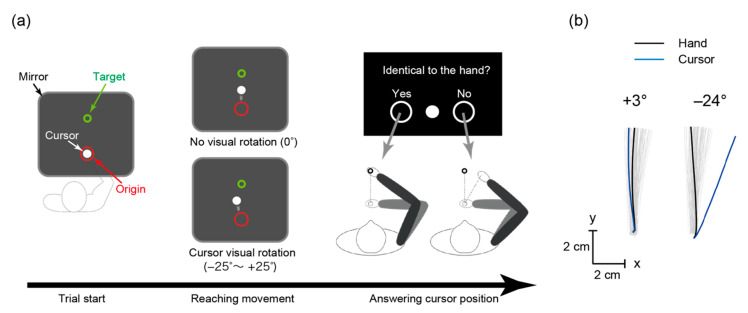
The arm-reaching task. (**a**) Participants moved their hand from the red origin towards the green target. Their hand and arm were not visible, and the white-cursor position could be visually rotated. After each trial, they answered whether the cursor and hand positions coincided by moving the handle towards the left or right. (**b**) Hand trajectories from a representative participant (gray lines), and examples of hand and cursor trajectories for small (+3°) and large (−24°) rotation angles.

We investigated sensorimotor integration, particularly in regard to the proprioceptive and kinesthetic ability to detect a gap, through the mean perception angle that the participants noticed as a gap between hand position and a visually-rotated cursor. The participants performed the reaching task with the right arm moving a white cursor shown on a computer display from a red circle (i.e., an origin point) towards a green circle (i.e., a target point) with the handle of the KINARM end-point manipulandum (BKIN Technologies, Kingston, Canada; see Figure 1). The handle and their arm were not visible as a mirror was positioned over them, through which the participants observed the display. For all trials, the target position was fixed at the coordinate of (1.7, 19.9) cm from the origin. They performed 108 trials wherein the cursor was visually rotated clockwise or counter-clockwise by 0–25° with increments of 1°. All rotation angles except 0° were selected once, in a random order. After each trial wherein the cursor was rotated, we inserted one or two trials without rotation to wash out after-effects. The participants answered whether the cursor position was identical to their hand position or not after every trial, with the distance being measured in a straight-line segment on the cartesian plane of the manipulandum, to which the display was co-registered. The target disappeared when the hand covered 2/3 of the distance towards the target (13.3 cm away from the origin) to ascertain that responses were mainly based on proprioceptive and kinesthetic sensation rather than visual information. This is particularly important to ensure that sensory reweighting, knowingly associated with aging, could not confound results via greater visual than proprioceptive reliance, even though other effects related to the visual component could not be entirely ruled out [30,31,32]. We recorded all responses using the KINARM manipulandum, which logged the rotation angles and cursor trajectories at sampling rate of 1000 Hz for all trials. Before the experiments, the participants completed 20 practice trials so as to become familiar with the task and the movement duration, which should be in the range of 300–500 milliseconds. A visual message “too fast” or “too slow” was displayed whenever the movement duration was outside that range. The manipulandum returned the hand to the origin after the reach was complete, and then the participant used the handle to answer whether the cursor and the hand’s position were identical (left) or not (right). Based on existing work, we assumed that individuals with high proprioceptive and kinesthetic performance would notice smaller discrepancies when a visual cursor displaying the position of the hand is displaced from its actual position [30]. As the participants actively moved their hand, the internal estimate of hand position is not only based on proprioceptive feedback but also the motor outflow or the efference copy of the motor commands, that is, aspects of kinesthesia [33]. Accordingly, the task did not index purely proprioceptive and kinesthetic sensitivity, in that it was also grounded on state estimation [34]; hence, the gap detection ability should be considered as an empirical measure.

### 2.3. Behavioral Data Analyses

From the visual rotation angles and corresponding participant responses in the arm-reaching task, we calculated the mean rotation angle of the incorrectly answered trials as the perception angle and used it as a proxy of individual gap detection ability. To evaluate the mean angle difference between the older and younger groups, psychometric curves were constructed for both groups, and the statistical difference was calculated using the threshold of 80% correct discrimination with a two-sample t-test. Subsequently, the mean perception angles were entered in the statistical analyses of resting-state connectivity data as covariates. In this study, the mean perception angles are referred to as “gap detection ability” with reference to the performance level in detecting non-zero rotation, which should not be confused with proprioceptive sensitivity in a strict sense, as the task involved active movement performance.

### 2.4. MRI Acquisition

To acquire the rsfMRI series, the structural volumes, and the 30-axis diffusion-tensor images, the consolidated and scanner-independent protocols of the Strategic Research Program for Brain Sciences were applied (https://bicr.atr.jp/decnefpro/) [35]. Due to magnetic resonance imaging (MRI) scanner replacement at the institute, the older and younger participants were studied, respectively, on a 3 T Signa scanner (General Electric Inc., Boston, MA, USA) and on a 3 T Magnetom Prisma scanner (Siemens AG, Munich, Germany). The temporal signal-to-noise ratio in the white matter during rsfMRI was not significantly different (108 ± 19 vs. 116 ± 15, *p* = 0.3), indicating comparability of the data. Large multi-centric studies have confirmed that, via the use of carefully harmonized acquisition protocols such as those adopted here, the confounding effect of inter-site and inter-scanner differences can be successfully controlled for functional, tractography and structural data [36,37,38].

Functional data were acquired, while the participants stared at a fixation circle, using a T2*-weighted gradient-echo echo-planar imaging sequence with the following parameters: repetition time (TR) = 2.5 s; echo time (TE) = 30 ms; flip angle (FA) = 80°; field of view (FOV) = 212 × 212 mm; matrix size = 64 × 64; 40 slices; slice thickness = 3.2 mm; 240 volumes. Furthermore, 30-directions diffusion tensor imaging (DTI) data were acquired using ASSET (in GE) and GRAPPA (in Siemens) sequence with the following parameters: for ASSET: TR = 16,000 ms; TE = 95.7 ms; FOV = 256 × 256 mm; matrix size = 128 × 128; 64 slices; slice thickness = 2.5 mm, for GRAPPA: TR = 14,100 ms; TE = 81 ms; FOV = 224 × 224 mm; matrix size = 114 × 114; 75 slices; slice thickness = 2 mm. For the anatomical MRI acquisition, a sagittal image was acquired using a T1-weighted spoiled gradient recalled sequence (for GE: TR = 7.7 ms; TE = 3.1 ms; FA = 11°; FOV = 260 × 260 mm; matrix size = 256 × 256; 200 slices; slice thickness = 1.2 mm, for Siemens: TR = 1.9 ms; TE = 2.52 ms; FA = 9°; FOV = 256 × 256 mm; matrix size = 256 × 256; 192 slices; slice thickness = 1.2 mm).

### 2.5. Regions of Interest (ROIs)

All image analyses focused on 6 sensorimotor ROIs bilaterally, as defined by the human motor area template (HMAT, [39]): the primary motor and sensory areas (M1, S1), SMA, pre-supplementary motor area (preSMA), dorsal and ventral premotor areas (PMd and PMv). The underlying rationale for these analyses was that the sensorimotor regions are collectively known to be implicated both in gathering proprioceptive afferences from the body and in using proprioceptive and kinesthetic feedback for regulating the performance of motor acts during activities such as walking and grasping, declining sensorimotor integration is therefore anticipated to possibly impact and be reflected by their interactions in manifold ways [17,22,40,41,42,43,44,45]. While other areas, such as S2 and posterior parietal structures, are also knowingly implicated in sensorimotor integration, they were not considered in this initial work due to the absence of consolidated, meta-analysis-based parcellation atlases comparable to the HMAT.

### 2.6. Functional and Effective Connectivity Analyses

Preprocessing of the rsfMRI images (i.e., slice-timing correction, spatial realignment, co-registration to the bias-corrected anatomical T1 image, calculation of normalization matrix to the Montreal Neurological Institute (MNI, Montreal, Quebec, Canada) standard brain, and smoothing with a Gaussian kernel of 6 mm full-width at half-maximum) and time-series extraction from the ROIs were performed using the CONN toolbox (https://www.nitrc.org/projects/conn) [46]. We entered rsFC intensities (Fisher transformed correlation coefficients) into analyses of covariance (ANCOVA) having one fixed factor for age, one covariate for gap detection ability (i.e., the mean perception angle), and the interaction term. The experimental hypothesis was evaluated through the statistical significance of the interaction. For brevity and to reduce the risk of confounding effects due to systematic scanner differences, the main effects of aging are not considered in detail.

For the region pairs showing significance in the functional connectivity analysis, we additionally queried the effective connectivity through stochastic dynamic causal modeling (DCM) [47].

### 2.7. Structural Analyses

We further calculated gray matter volumes through voxel-based morphometry (VBM) analysis based on SPM12, and diffusional characteristics (i.e., fractional anisotropy and diffusivity) on axonal bundles through DTIfit (https://fsl.fmrib.ox.ac.uk/fsl/fslwiki/FDT/UserGuide#DTIFIT) and probabilistic fiber tractography based on ProbtrackX [48] to investigate whether these functional effects were accompanied by structural changes. The latter analyses were performed individually for the region pairs corresponding to all rsFCs that showed significance, with 1 participant rejected due to data corruption. For each normalized brain, a way-point probabilistic tractography was conducted setting independently each of the bilateral pre-SMA ROIs as seed masks and the corresponding ipsilateral SMA as a target. A similar approach was followed for the other region pairs. To reconstruct the resulting tracts, a threshold of 40% minimum probability was used, with fractional anisotropy and diffusivity averaged over the entire resulting volume.

## 3. Results

### 3.1. Behavioral Difference between Younger and Older Adults

**Figure 2:**
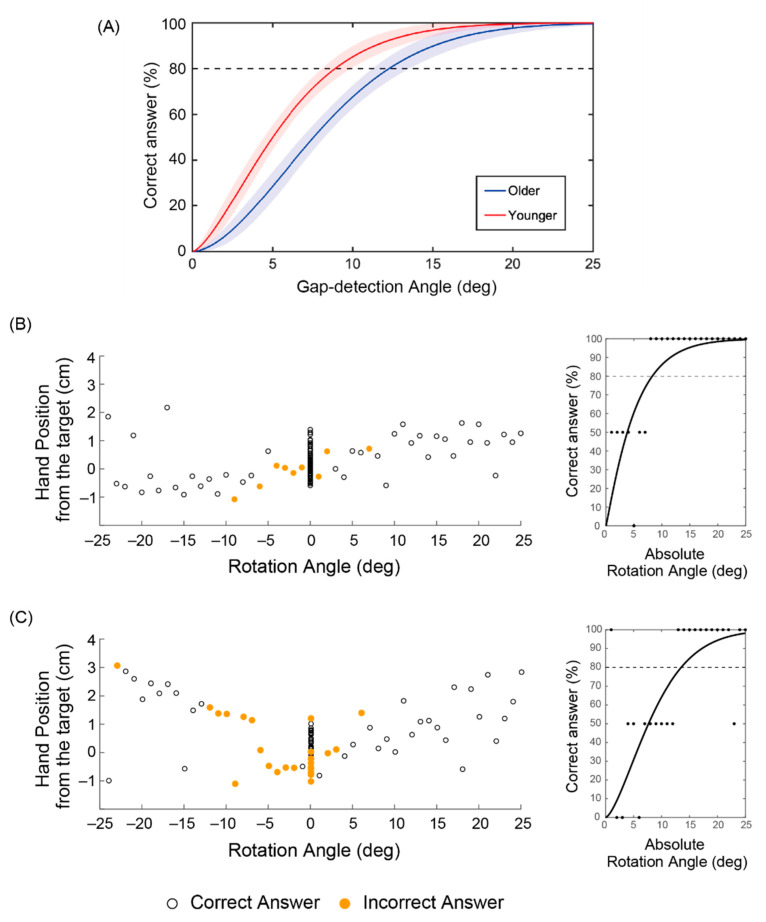
Reaching task results. (**a**) Correctly perceiving a discrepancy in the cursor position as a function of the gap-detection angle between older and younger groups. Psychometric curves with 95% confidence interval fitted using Weibull functions to the pooled group data, wherein the dotted line denotes the threshold of 80% correct discrimination. (**b**) and (**c**) Examples of younger and older representatives, respectively. Reaching hand position (*y*-axis) and cursor rotation angles (*x*-axis) plotted with correct and incorrect answers in the left panels together with individual fitted curves in the right panels.

To confirm the efficacy of the arm-reaching task in investigating proprioceptive and kinesthetic sensitivity as empirically indexed by the gap detection ability, we compared the psychometric curves between the younger and older groups in Figure 2a [30]. Predicated on the assumption that even a small decrease can seriously impact daily living in older age, for example, in regard to falls, we compared the mean angles at 80%-correct probability levels: these were 11.1 ± 2.5° (mean ± standard deviation) for the older and 8.3 ± 3.2° for the younger participants (*p* = 0.04, two-sample t-test). Figure 2b,c shows examples of younger and older participants, respectively. As expected, younger participants failed to notice smaller rotation angles (Figure 2b), whereas older participants could misclassify larger rotation angles >20° (Figure 2c). Notably, the participants with lower gap detection ability displayed larger variance in the hand’s position when reaching towards the target.

The results pointed to the value of the arm-reaching task in evaluating sensorimotor integration, particularly in regard to proprioceptive and kinesthetic sensitivity. For the following analyses, we took the mean rotation angle of the incorrectly answered trials with rotation as a simple empirical index of gap detection ability and used it to search for rsFCs associated with age-related decline, since the mean rotation angle was considered more direct and proper as compared to the rotation angle derived from the psychometric curves, which involves fitting a function. This parameter was also different between the older and the younger participants (6.5 ± 2.2° vs. 4.5 ± 1.8°, *p* = 0.04), and the accuracy in classifying trials with and without rotation was higher in the latter (0.78 ± 0.05 vs. 0.84 ± 0.06, *p* = 0.02).

### 3.2. Functional Connectivity Reflecting Age and Gap Detection Ability

**Figure 3:**
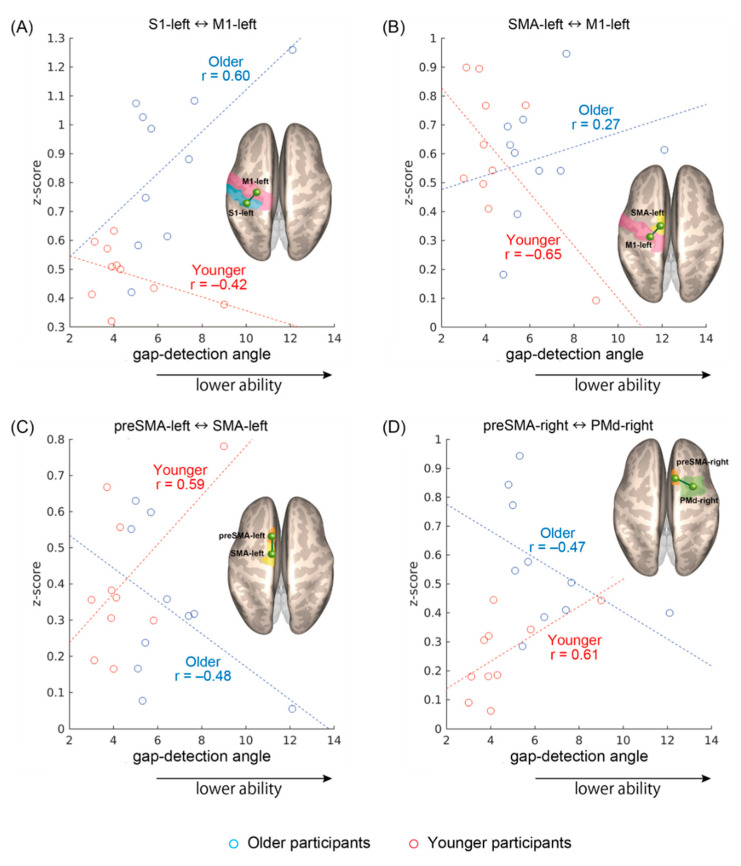
Significant resting-state functional connectivity (rsFC) showing specular interaction effects between age and gap detection ability. (**A**) Connectivity between the left primary sensory area and the left primary motor area (S1left–M1left), (**B**) the left supplementary motor area and the M1left (SMAleft–M1left), (**C**) the left pre-supplementary motor area and the SMAleft (preSMAleft–SMAleft), and (**D**) between the right pre-supplementary motor area and the right premotor area (preSMAright–PMdright). Pearson correlation coefficients are given regardless of the significance of individual regressions.

As shown in Figure 3a–d, four rsFC showed significant interaction effects between age and gap detection ability: S1left–M1left (*F*(1,16) = 5.2, *p* = 0.04), SMAleft–M1left (*F*(1,16) = 5.5, *p* = 0.03), preSMAleft–SMAleft (*F*(1,16) = 6.6, *p* = 0.02), and preSMAright–PMdright (*F*(1,16) = 5.5, *p* = 0.03). The sign of the correlations was consistently different between the age groups: the S1left–M1left and SMAleft–M1left rsFCs (Figure 3a,b) tended to become stronger as gap detection ability declined in the older participants, whereas the opposite trend was apparent in the younger participants. On the other hand, in the preSMAleft–SMAleft and preSMAright–PMdright rsFCs (Figure 3c,d), stronger connectivity tended to be observed in the younger participants with lower gap detection ability and older participants with higher gap detection ability. For S1left–M1left and preSMAright–PMdright, the rsFC was on average stronger in the older participants, with additional and heterogeneous group differences seen for other region pairs but no overall effects of performance.

The results were additionally confirmed with a non-parametric approach. Namely, for each linear regression, the corresponding Spearman rank-order correlation coefficient was calculated. All signs remained unaltered, yielding, respectively, in the younger and older groups, *r* = −0.22 and *r* = 0.56 for S1left–M1left, *r* = −0.38 and *r* = 0.27 for SMAleft–M1left, *r* = 0.28 and *r* = −0.37 for preSMAleft–SMAleft, and *r* = 0.64 and *r* = −0.62 for preSMAright–PMdright. However, because of the small sample size in this preliminary work, the individual correlations did not reach statistical significance, only documenting the opposite trends underlying the interaction effects.

On the other hand, entering the rotation/no-rotation classification accuracy rate instead of the gap detection ability in the analyses, no significant interactions were found, despite the presence of a marked group-level behavioral difference. Considering the sum rather than the mean rotation angle of the incorrectly answered trials and thus indexing a cumulative effect, analogous interactions remained for the SMAleft–M1left (*F*(1,16) = 4.6, *p* = 0.047) and preSMAleft–SMAleft (*F*(1,16) = 6.5, *p* = 0.02).

### 3.3. Effective Connectivity Reflecting Age and Gap Detection Ability

We additionally queried the effective connectivity according to a DCM model encompassing S1left, M1left and SMAleft in a clique alongside the chain SMAleft–preSMAleft–preSMAright–PMdright; here, preSMAleft–preSMAright was included out of anatomical considerations, even though no significant rsFC interaction was present. Two of the four rsFCs involving the preSMA showed interaction effects on their bidirectional couplings, namely preSMAleft→SMAleft (*F*(1,16) = 6.3, *p* = 0.02), SMAleft→preSMAleft (*F*(1,16) = 6.4, *p* = 0.02), PMdright→preSMAright (*F*(1,16) = 6.0, *p* = 0.03), and preSMAright→PMdright (*F*(1,16) = 6.8, *p* = 0.02). Additionally, a unidirectional effect was found for preSMAleft→preSMAright (*F*(1,16) = 5.4, *p* = 0.03).

### 3.4. Structural Aspects Reflecting Age and Gap Detection Ability

Although the six areas (i.e., S1left, M1left, SMAleft, preSMAleft, preSMAright, and PMdright) all showed significant age-related grey matter volume loss, only the SMAleft displayed an age-by- gap detection ability interaction (*F*(1,16) = 5.1, *p* = 0.04), wherein the volume was larger in the younger participants with lower gap detection ability (*r* = 0.70) but insensitive in the older participants. Adjusting for intracranial volume had no impact.

**Figure 4:**
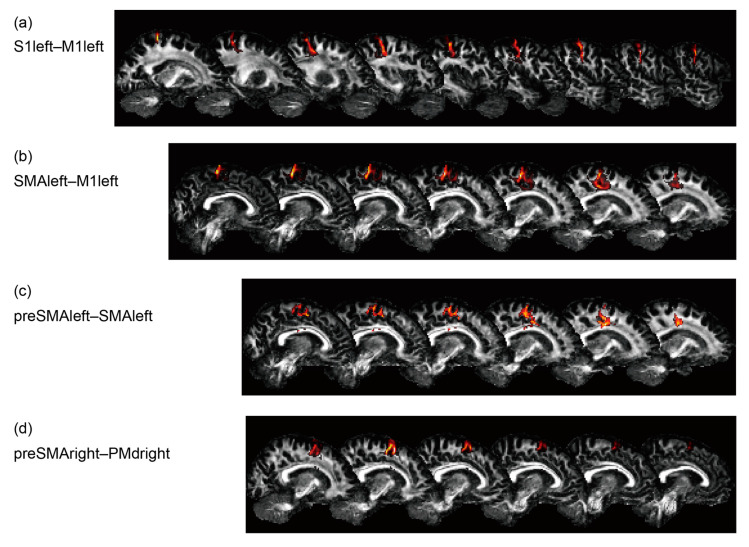
Reconstruction results of probabilistic tractography for a representative participant. (**a**) S1left–M1left, (**b**) SMAleft–M1left, (**c**) preSMAleft–SMAleft, and (**d**) preSMAright–PMdright.

Probabilistic tractography was performed for the region pairs corresponding to all four rsFCs, S1left–M1left, SMAleft–M1left, preSMAleft–SMAleft, and preSMAright–PMdright, as shown in Figure 4. It did not reveal any interactions for mean diffusivity and fractional anisotropy, though the axonal bundles between S1left–M1left (*F*(1,15) = 6.1, *p* = 0.03) and preSMAright–PMdright (*F*(1,15) = 4.6, *p* = 0.049) had increased diffusivity in the older participants.

## 4. Discussion

In this exploratory study, we hypothesized that the decline in sensorimotor integration, and particularly proprioceptive and kinesthetic sensitivity with aging, involves the sensorimotor brain networks, and investigated the hypothesis in a task-independent manner using rsfMRI. Being consistent with previous work [30], the arm-reaching task also showed the significant difference of gap detection ability between the younger and older participants. The correlation analyses to find rsFC that showed significant interaction effects between age and the gap detection ability revealed four connectivities: S1left–M1left, SMAleft–M1left, preSMAleft–SMAleft, and preSMAright–PMdright. These results suggest that the decline of performance is represented in sensorimotor brain networks of resting-state brain activity.

Due to the small sample size and sex bias, future confirmation in larger samples is required, particularly in regard to the significance of the individual correlations. Conservatively taking the weakest Pearson correlation observed as reference to reach definite conclusions, namely *r* = 0.27, post-hoc power analysis assuming α = 0.05 indicates a sample size of 140 participants per group, which should be sex balanced. Nevertheless, the present results were corroborated both by parametric and non-parametric approaches, and the other performance parameter (i.e., rotation/no-rotation classification) did not show significant interaction effects in the four connectivities. Although the reaching task used in this study may reflect not only kinesthetic and proprioceptive error but also prediction error [30,33], this corroborates the view that the observed neural effects could be reflective of sensorimotor integration rather than of broader cognitive aspects influencing task performance. While the matter remains incompletely known, age-related proprioception decline appears to be influenced by different factors rather than general cognitive changes [49].

In the subsequent effective connectivity analysis, two rsFCs involving the preSMA, namely preSMAleft–SMAleft and preSMAright–PMdright, showed significance. The results reaffirmed the rsFC observations through a different and model-centric computational approach. The morphometry analysis showed an age-by-gap detection ability interaction in SMAleft. The volume was larger in the younger participants with lower gap detection ability but insensitive in the older participants. This association may reflect inter-individual differences, and it cannot be excluded that it could also affect the correlations shown in Figure 3b,c. Nevertheless, considering that no interactions were found in mean diffusivity and fractional anisotropy, it can be asserted that the impact of aging on the gap detection ability at the cortical level seems to be primarily functional, rather than structural.

**Figure 5:**
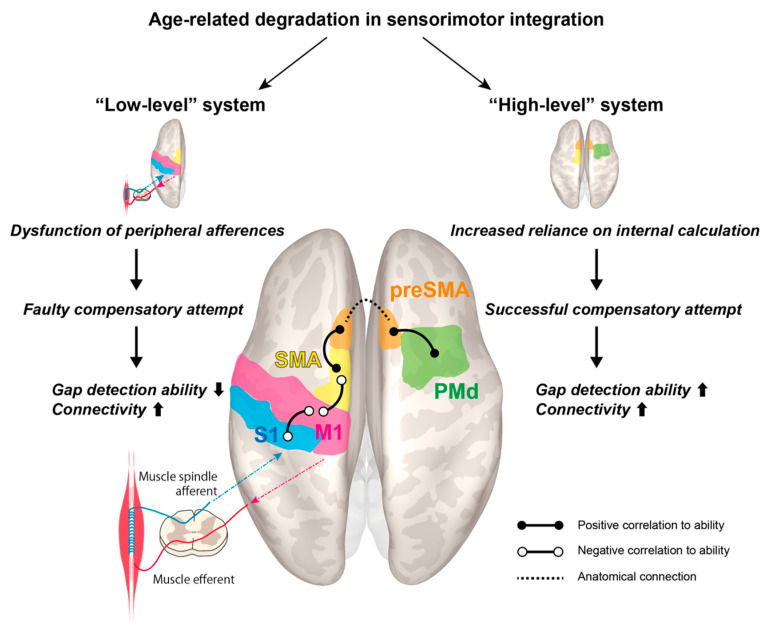
Proposed model for cortical reorganization accompanying age-related decline in sensorimotor integration.

Based on the experimental results, we offer a hypothetical model, dubbed the “dual-system model”, which appears worthy of future investigation in larger cohorts (Figure 5). It revolves around the schematized notion of an age-related decline in a “low-level” system, which is partly compensated via greater engagement of “high-level” regions, in tune with previous observations that declining proprioceptive peripheral inputs are accompanied by enhanced centrally-generated signals; this model is well in line with previous results obtained from the application of substantially different approaches [17]. This somewhat traditional distinction between “low-level” and “high-level” regions is retained for lexical convenience, though recent research points away from a strictly hierarchical view of the motor cortices [50,51].

The well-established age-related loss in peripheral afferences, particularly from muscle spindles as observed in rat models [52], is a plausible causative factor for the observed overconnectivity between S1left–M1left and SMAleft–M1left. Nonetheless, the compensatory attempt is ultimately unsuccessful in this “low-level” system, leading to a paradoxical correlation with reduced gap detection ability. On the other hand, in the other two rsFCs, preSMAleft–SMAleft and preSMAright–PMdright, a successful compensatory attempt plays out, such that older individuals with high gap detection ability feature boosted connectivity. We hypothesize that they may rely more heavily on this “high-level” system, which produces internal estimations of body dynamics tracking the end effectors. While we cannot offer a conclusive explanation for the lateralized effects, it should be noted that the left hemisphere corresponds to the dominant side. Although we did not detect an interaction between preSMAleft–preSMAright, these regions are knowingly connected inter-hemispherically.

In the proposed model, the SMA plays the pivotal role of a “gateway station” between the posited “low” and “high-level” systems. Throughout the existing studies on motor aging, this is the structure for which significant effects have been most prominently reported. This plausibly reflects its central role in coordination, and in the dense connectivity within and outside the motor system, and its functional differentiation, particularly between its posterior and anterior part—the so-called pre-SMA [53,54,55]. Aging is associated with sensorimotor attenuation, resulting in increased reliance on internal predictions generated in the pre-SMA [56]. Previously identified interactions between age and task difficulty point to weaker facilitation of motor performance [57,58], and alterations of functional and effective connectivity have been found for the SMA and pre-SMA in the elderly [43,44]. Furthermore, age-related de-differentiation in the motor system is well established [40,59].

Our study, while limited in its conclusiveness by the small study sample, provides a testable, compact, and simple model that systematizes these observations in a straightforward experimental paradigm with potential for clinical application. This is underlined by previous observations that, in the elderly, the SMA responds differently to the individual’s difficulty during walking-while-talking, indicating its involvement in age-related motor impairments that lead to falls and other accidents [41,60]. In aging cohorts, the SMA activity and morphology correlate with gait speed and postural instability [42,61] and, consequently, fear of falling [45]. Our experiment specifically addresses visuo-motor control, which represents a fundamental aspect of vulnerability in real-life settings. Albeit still exploratory, the present results underline the importance of coordination exercise for maintaining central motor function [62], extending previous findings about the benefits of motor training on SMA connectivity [63].

## 5. Conclusions

In this exploratory study, we investigated the role of cortical motor networks under a hypothesis that the age-related decline in sensorimotor integration, particularly the proprioceptive and kinesthetic sensitivity, is correlated with differences in the connectivity between and within the sensorimotor areas. The gap detection ability measured during the arm-reaching task showed a significant difference between the younger and older participants. The correlation analyses to find rsFC showed significant interaction effects between age and the gap detection ability in the S1left–M1left connection, SMAleft–M1left, preSMAleft–SMAleft, and preSMAright–PMdright. As morphometry and tractography analyses did not show corresponding structural effects, these preliminary results suggest that the decline of performance is represented in the form of functional activity of sensorimotor brain networks in the resting-state.
